# Role of N-glycosylation in activation of proMMP-9. A molecular dynamics simulations study

**DOI:** 10.1371/journal.pone.0191157

**Published:** 2018-01-12

**Authors:** Sonu Kumar, Piotr Cieplak

**Affiliations:** SBP Medical Discovery Institute, La Jolla, California, United States of America; Chang Gung University, TAIWAN

## Abstract

Human matrix metalloproteinase proMMP-9 is secreted as latent zymogen, which requires two-steps proteolytic activation. The secreted proMMP-9 is glycosylated at two positions: Asn38 and Asn120 located in the prodomain and catalytic domain, respectively. It has been demonstrated that glycosylation at Asn120 is required for secretion of the enzyme, while the role of Asn38 glycosylation is not well understood, but is usually linked to the activation process. One hypothesis stated that the Asn38 glycosylation might protect against proteolytic activation. However, the activation process occurs with or without the presence of this glycosylation. We conducted molecular dynamics (MD) simulations on the glycosylated and non-glycosylated proMMP-9 to elucidate the effect of Asn38 glycosylation on this two-step activation process. The simulation results suggest that Asn38 glycosylation does not hinder the activation process directly, but induces conformational changes in the vicinity of the first proteolytic region in such a way that E^59^-M^60^ cleavage is processed before R^106^-F^107^. These results correlate with analysis provided by Boon et al. and experimental data from Ogata et al. who attempted to determine the order of events in activation of proMMP-9. Results from additional MD simulations for the model of glycosylated proMMP-9 bound to galectin-8 N-domain suggest that Gal-8 by interacting with Asn38 glycan might further facilitate processing of the first cleavage between E^59^-M^60^. Thus, our simulation results suggest that both Asn38 glycosylation and interaction with Gal-8N may be involved in facilitating and the temporal order of the activation process of pro-MMP9. The aim of this report is to provide an inspiration for future detailed experiments aimed at explaining the role of N-glycosylation in the activation process of prodomain of MMP-9.

## Introduction

Matrix metalloproteinase-9 (MMP-9), also known as gelatinase B, is a Zn^+2^–dependent metalloendopeptidase [1] that is capable of degrading many extracellular matrix components. It plays an important role in normal tissue remodeling and pathological degradation of the extracellular matrix in several human autoimmune diseases, cancer metastasis, diabetes, and arthritis [2–4].

MMP-9, like other MMPs, is secreted from a cell as latent zymogen proMMP-9 and requires proteolytic activation in which the inhibitory prodomain is removed and the catalytic domain is exposed for its substrate’s proteolytic processing. MMP-9 is tightly regulated at the expression level and also by endogeneous inhibitors such as tissue inhibitors of metalloproteinase (TIMP) [5, 6]. A number of purified proteases, including trypsin [7, 8], chymase [9], MMP-2 [10], tissue kallikrein [11], trypsin-2 [12], plasmin [13, 14], MMP-7 [15], MMP-13 [16], and MMP-3 [7–9, 13, 14, 17, 18] have been reported to activate proMMP-9 in vitro. But based on in vitro kinetic and catalytic parameters, MMP-3 appears to be most efficient activator of proMMP-9 [7] and may be even a natural activator in vivo. It is also reported that proMMP-9 is activated by MMP-3 (stromelysin-1) in a stepwise manner. MMP-3 initially cleaves proMMP-9 at the E^59^-M^60^ located in the middle of the prodomain. This proteolytic event triggers conformational change in proMMP-9 which exposes the R^106^-F^107^ peptide bond to the MMP-3 for the second cleavage event [17]. This stepwise activation mechanism is similar to that seen in other members of the MMP family such as MT1-MMP (MMP-14) [19], however it is performed by different activating enzymes.

The full-length proMMP-9 is a glycoprotein with three N-glycosylation and multiple O-glycosylation sites. In general, proteins are glycosylated for various mechanistic reasons such as promotion of proper protein folding, recognition of improperly folded proteins and their degradation, sorting events and perhaps protection from proteases after secretion [20]. In proMMP-9 there are three possible N-glycosylation sites Asn38, Asn120, and Asn127, identified by the canonical NxS/T sequence motif. However, it was experimentally determined that only two of them have been actually glycosylated. One glycan is located at Asn38 in the inhibitory prodomain, and the other is attached to Asn120 in the catalytic domain. The N-glycosylation at Asn127 has not been observed experimentally, most likely due to the steric hindrance exerted by the fibronectin domain. Both the glycosylation sites are populated by NeuAcα(1,2)-Galβ(1,4)-GlcNAcβ(1,2)-Manα(1,3)-[NeuAcα(1,2)-Galβ(1,4)-GlcNAcβ(1,2)-Manα(1,6)-]Manβ(1,4)-GlcNAcβ(1,4)-[Fucα(1,6)-]GlcNAcβ-Asn glycan chain [21]. Additionally, in proMMP-9 there are multiple O-glycosylation sites located in the O-link connecting the catalytic and hemopexin domains. At the cellular level, more than 95% of the N-linked glycans attached to proMMP-9 are partially sialylated, core-fucosylated biantennary structures, with or without the α1,6 fucosylation branch. The O-linked glycans comprise approximately 85% of the total sugars on proMMP-9, mainly of type 2 cores with lactosamine (Galα1,4GlcNAc) extensions, with or without sialic acid or fucose [22]. The truncated proMMP-9 structure consists of the inhibitory prodomain, the catalytic domain, and three-fibronectin type II domains that are shown in S1 Fig.

Interestingly, another member of the gelatinase family, proMMP-2 is quite similar in structure to proMMP-9 and is also involved in the cleavage of denatured collagen. However, it lacks N-glycosylation sites and has a fewer number of putative O-glycosylation sites compared to proMMP-9, which is an intriguing observation. The stabilizing effect provided by these glycosylation to proMMP-9, in contrast with proMMP-2, likely enables it not to be readily activated in cellular systems. This has been confirmed by experimental work of Kotra *et al*. [21], who demonstrated that purified proMMP-9 exhibited resistance to autocatalytic activation in solution. The effect of glycosylation on MMP-9 and other members of matrix metalloproteases and other enzymes has been studied by several authors, see for example the references [22–25]. The research area focused on glycosylation of matrix metalloproteases has been recently reviewed by Boon *et al*. [26].

Considering the role of glycans in full-length proMMP-9, Nishi *et al*. [27] found that galectin-8 accelerated MMP-3 mediated processing of proMMP-9 in solubilized neutrophil membrane by making a ternary complex. Galectin-8 (Gal-8) consists of the N- and C- domains connected by a linker sequence of various lengths [28, 29]. The N-domain of Gal-8 recognizes sialylated LacNAc [30], whereas the C-domain recognizes lactose or LacNAc [31]. It has been found that the N-domain of Gal-8 interacts with carbohydrate of proMMP-9, while the C-domain interacts with both integrin αM/CD11b and proMMP-9, which enhances MMP-3 mediated processing of proMMP-9 [27]. These interaction patterns and the role of glycans in the binding within the ternary complex are still not well-understood phenomena at the molecular level. The most recent report describing interaction between glycosylated form of MMP-9 and another member of the galectin family, galectin-3 has been published in 2006 by Fry *et al*. [23].

Recently it was reported that proMMP-9 with the N38S mutation in prodomain is efficiently secreted, whereas the N120S mutation reduces the level of proMMP-9 secretion [25]. This suggests that Asn120 glycosylation is essential for the secretion process, while Asn38 glycosylation may be involved in controlling processes that take place after the secretion, such as activation of the pro-enzyme.

Despite this background information regarding glycosylation in proMMP-9, the role of the prodomain N-glycosylation is unclear. We sought to probe whether the function of glycosylation is to protect proMMP-9 against proteolytic action of activating MMP-3. We investigated this problem at the structural level using MD simulations. The results of simulations suggest that the complex N-glycan at Asn38 may not protect the proteolytic region from being processed. Instead, we observed that this glycosylation may be responsible for inducing conformational changes in the region of E^59^-M^60^ cleavage that prevent the other R^106^-F^107^ cleavage from being processed first. Thus, we hypothesize that the proMMP-9 activation mechanism might be a two-step process that is orderly regulated by the Asn38-glycosylation in the prodomain.

## Results and discussion

### Molecular dynamics of glycosylated and non-glycosylated forms of proMMP-9

**Fig 1:**
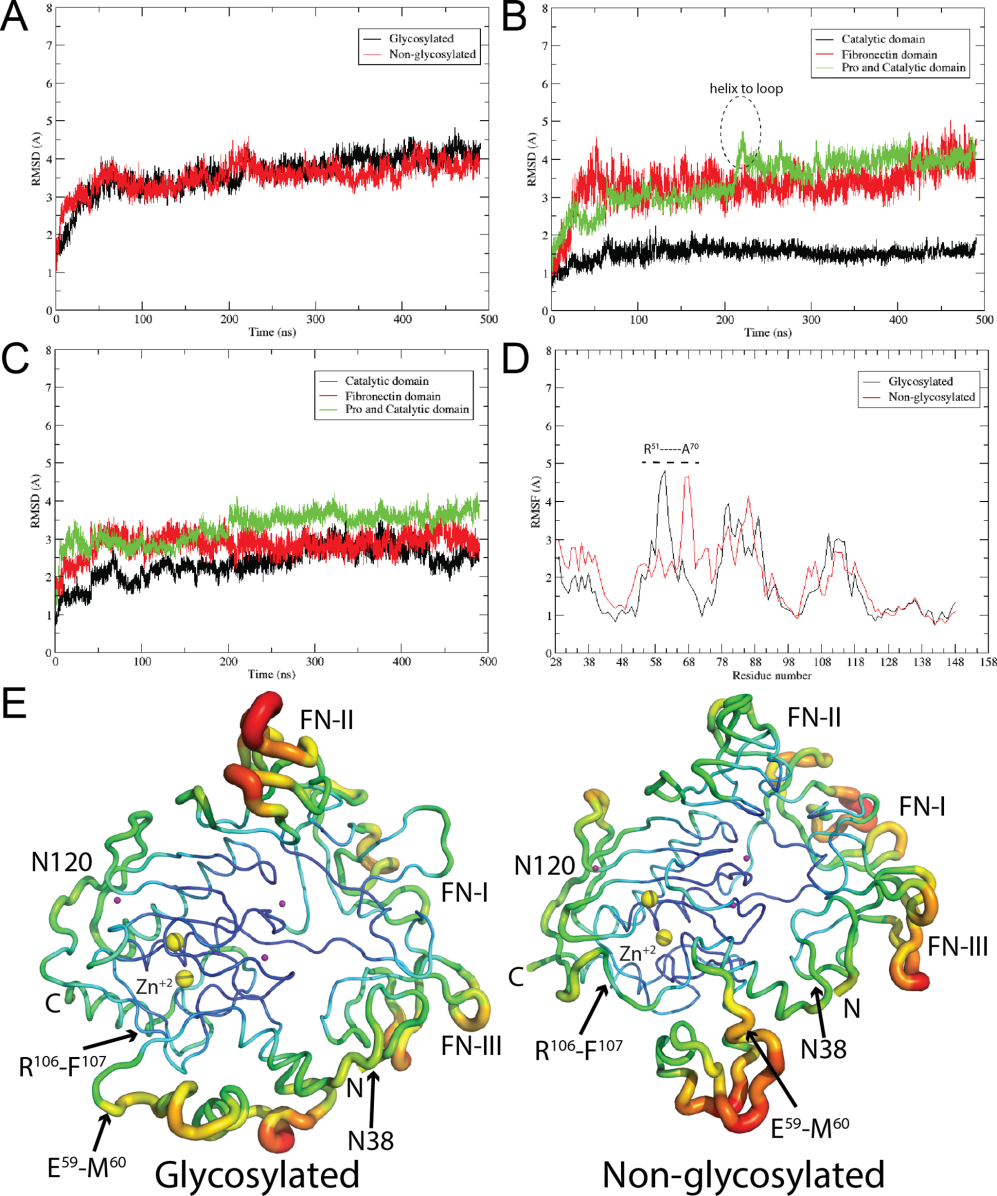
Analysis of the RMSDs (Cα atoms only), RMSFs, and B-factors of the glycosylated and non-glycosylated forms of proMMP-9. (A) Comparison of RMSDs of proMMP-9. (B) Comparison of RMSDs of the catalytic domain, the fibronectin domain, and the pro and catalytic domains together for glycosylated form of proMMP-9. (C) Comparison of RMSDs of the domains listed in (B), for non-glycosylated form of proMMP-9. (D) Comparison of RMSFs for prodomain in glycosylated and non-glycosylated form of proMMP-9. Significant changes in region R51-A70 are marked with dashed line. (E) Represents the displacement of the atomic positions from an average value (B-factor) of the glycosylated and non-glycosylated form of proMMP-9. The catalytic zinc ion is marked as yellow sphere and labelled Zn^+2^.

To understand the structural role of glycosylation in MMP-3 mediated activation of proMMP-9, we performed MD simulations for non-glycosylated and glycosylated forms of the proMMP-9 structures in aqueous solution. As a first step in analysis of MD trajectories we inspected stability of the protein structures during MD simulation by calculating the root mean square deviation (RMSD) from the initial structures as a function of time. The appropriate RMSD plots for backbone atoms for full proMMP-9 are presented in Fig 1A. They demonstrate that simulations are stable throughout the entire MD simulations. The slow ascending behavior of the RMSDs is associated with conformational fluctuations localized in subdomains of fibronectin and prodomain. The corresponding RMSD plots for atoms belonging either to the catalytic domain, fibronectin domain or both the pro- and catalytic domain region are shown in Fig 1B and 1C, for glycosylated and non-glycosylated forms of pro-MMP9, respectively. The RMSDs for the catalytic domain for glycosylated form (black line, Fig 1B) is substantially lower (1.5 Å) compared to non-glycosylated (black line, Fig 1C) form of the enzyme (2.5–3.0 Å). Thus, glycosylation may be considered as having a stabilizing effect on the catalytic domain. The role of glycosylation could be further discussed in terms of root mean square fluctuations (RMSF) for each residue over the entire trajectory. Appropriate RMSFs for all amino acids (residues: 28–445) and those belonging to the prodomain region (residues: 28–112) are shown in S2 Fig and Fig 1D, respectively. The results indicate higher degree of flexibility in prodomain of the non-glycosylated proMMP-9 as compared to glycosylated proMMP-9. We use Kolmogorov-Smirnov test for comparing the distribution of RMSFs values calculated using only C-α atoms of each residue for both glycosylated and non-glycosylated cases. The C-α atom RMSF distribution for the non-glycosylated form was significantly greater than for the glycosylated form of proMMP-9 (distance D = 0.2083, p-value = 0.011, which is lower than the threshold p < 0.05 in Kolmogorov-Smirnov test), indicating that glycosylation changes the dynamics of the prodomain backbone. This is also illustrated by calculating the displacement of the atomic positions from an average value (B-factor) for the protein backbone atoms. In Fig 1E the flexibility of the protein backbone is depicted from high to low B-factor values by red to blue shades and thickness of the ribbon, respectively. The highest degree of flexibility is observed for fibronectin domain and the prodomain. For the prodomain higher flexibility is exhibited for the non-glycosylated proMMP-9 compared to its glycosylated form, particularly in the vicinity of the first cleavage site. This observation supports notion that glycosylation contributes to the stabilization of the protein structure.

**Fig 2:**
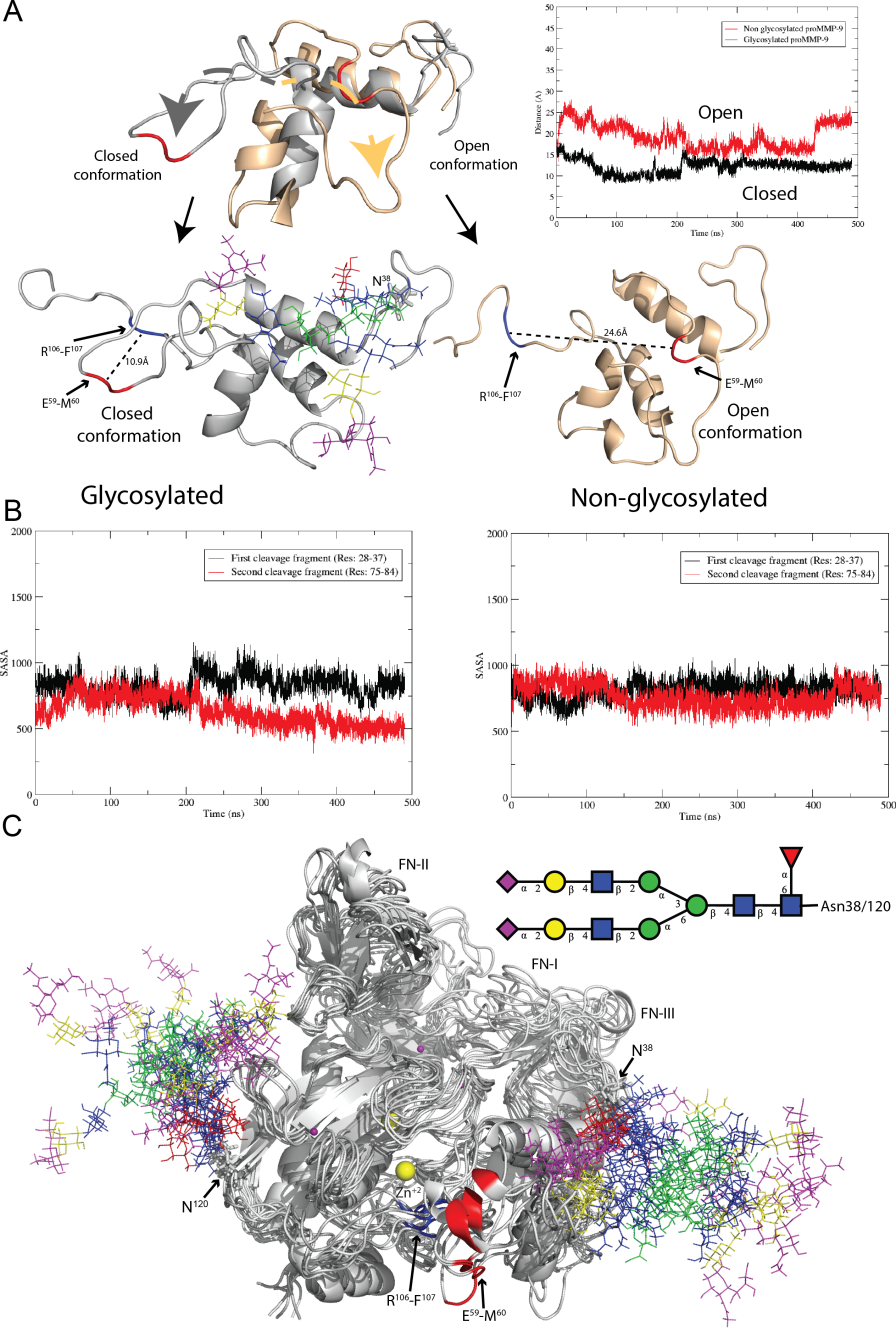
Illustration of the conformational changes occurring in the proteolytic region of the prodomain, solvent-accessible surface area and multiple structure alignments. (A) Superimposition of the prodomain proteolytic region (top panel) shows significant conformational changes (R51-A70) in the glycosylated in contrast to non-glycosylated proMMP-9. The right top panel represents times-series of the distance between the Cα atoms of E59-R106 in the glycosylated (black) and non-glycosylated (red) proMMP-9, demonstrating the mobility of the loop. The lower left panel represents the glycosylated proMMP-9 prodomain in closed conformation (first cleavage region protects the second one and the distance between them is 10.9 Å). Lower right panel represents the non-glycosylated proMMP-9 prodomain in open conformation (both cleavages regions are exposed and the distance between them is 24.6 Å). (B) Left and right panel represents the solvent-accessible surface area (SASA in Å^2^) of two proteolytic fragments for glycosylated and non-glycosylated form of proMMP-9, respectively. (C) Superimposition of seven snapshots, each at 80ns intervals out of the 500ns MD trajectory. Glycans are represented according to the Consortium For Glycomics nomenclature.

In this section, we will discuss the conformational changes which take place in the amino acid sequence between R^51^-A^70^ during MD simulations. The discussion will be illustrated using only one trajectory, however the similar behavior has been observed in two independent simulations for glycosylated form of proMMP-9 and in simulations involving the complex with Gal-8. During MD simulations, we observe substantial conformational changes in the vicinity of the first activating cleavage site. This region spans R^51^YGYTRVAEMRGESKSLGPA^70^ portion of the proMMP-9 prodomain sequence. The glycosylation at Asn38 induces conformational changes in this region in such a way that the second, R^106^-F^107^ proteolytic site becomes shielded from the activating MMP-3 enzyme. This conformational rearrangement occurred during the initial 5ns of the MD simulation after which the structure becomes stable. The only further rearrangement of helix to loop in the vicinity of the first cleavage site for the case of glycosylated proMMP-9 is observed around 230 ns of MD simulations as shown in Fig 1B. This conformational change could be quantified by measuring the distance between the first and second cleavage sites. In the “Closed” conformation, where the second cleavage site is shielded, this distance is ≈11 Å. In contrast, in the proMMP-9 without glycosylation at the Asn38, the amino acid sequence between positions 51–70 tends to stay on the other side of the triple helical motif, located away from the second proteolytic site. In this “Open” conformation the distance between the first and second cleavage sites is ≈25 Å) (Fig 2A). The distance between the CA atoms of E59-R106 in both the glycosylated and non-glycosylated proMMP-9 are represented in Fig 2A, right top panel, illustrating the motion of the loop, forming either “Closed” or “Open” conformation. In previous experimental studies it has been demonstrated that proMMP-9 activation is an ordered two-step process [17], however the detailed mechanism of this event has not been revealed. According to our calculations when the first cleavage event between E^59^-M^60^ residues occurs, the prodomain changes its conformation and exposes the second cleavage site, between R^106^-F^107^, for final activation. Results of our simulations also indicate that the glycan at Asn38 in proMMP-9, due to its internal dynamics, is unable to directly shield either of the two cleavage sites from the activating MMP-3 enzyme. Thus, one would expect that both sites should be equally accessible for proteolysis, with some preference for the cleavage at R^106^-F^107^ peptide bond, because it is more exposed to the solvent than the E^59^-M^60^ site, in the initially built structure based on non-glycosylated form (see solvent-accessibility surface area (SASA) of both proteolytic fragments in glycosylated form of proMMP-9, left panel of Fig 2B). In the case of glycosylated proMMP-9 the Asn38 glycosylation, in indirect manner alters the conformation of the prodomain in such a way that the R^106^-F^107^ proteolytic site is protected from being processed first.

Another member of gelatinase family MMP-2 is also activated in a two-step process, however, its prodomain is not N-glycosylated. Activating MT1-MMP (MMP-14) enzyme initially cleaves proMMP-2 at the N^37^-L^38^ located in the middle of the prodomain and generates the intermediate 68KDa product [32]. The second cleavage N^80^-Y^81^ peptide bond, which is mediated by active MMP-2 in an autocatalytic manner in *trans* [32, 33], generates a fully active 66KDa MMP-2. In this two-step activation process, the second step is prevented to occur first by the mechanism involving exogenous MMP-2 hemopexin C-domain. It is suggested that this process needs to be mediated by membrane localization rather than a soluble, active MMP-2 molecule [34]. In summary, even though both MMP-2 and MMP-9 belong to the same gelatinase family their mechanism of activation is different.

In Fig 2C we present seven superimposed snapshots from MD simulations, at 80ns intervals from each other, to demonstrate the range of glycan movement at both Asn38 and Asn120 glycosylation sites. Each carbohydrate type is represented in its standard CFG color to better understand the range of movement of each particular residue. A high degree of flexibility of glycan at both N-glycosylation sites can be observed. Yet, despite this large glycan flexibility, the glycan at Asn38 is unable to effectively shield the first cleavage site to protect it against proteolytic activity of MMP-3. A model of the activating complex of glycosylated MMP-9 and MMP-3 is shown in S3 Fig. However, this model built structure has not been used for simulations because it is provided only for illustration and accuracy of this model cannot be tested. During the first 230ns MD simulations of glycosylated proMMP-9 the first proteolytic region adopts a helical structure but beyond this time point it turns into a loop and maintains this form until the end of the simulation (at 500ns). This secondary structure transition occurs between 220–230 ns portion of the MD trajectory (marked as “helix to loop” in Fig 1B). After this event, the structure remains stable and no further substantial secondary structure change is observed. We demonstrate this transition in S4 Fig, by calculating occupancy of the secondary structure [helix (alpha, 3–10, pi), sheet (parallel, anti), and loop (turn)] during 500ns trajectory. The first proteolytic region (E^59^-M^60^) predominantly adopts loop conformation (50–60% of the time), but contribution from alpha helical form (less than 20%) is also observed (S4 Fig).

The other complex N-glycan located at the Asn120 position also demonstrates a high degree of flexibility. It is positioned far from the catalytic region and both the proteolytic sites, however, it plays important role in the secretion process as shown by Duellman et al. [25]. Our simulations suggest that this glycan affects the dynamics of the fibronectin domain by interacting with one of its subdomains (Fig 2C). In overall, it is expected that glycosylation at Asn120 does not interfere with the catalytic process, mainly because it is located further away from the catalytic binding site. Also, it is clear, that despite of a putative glycosylation motif being present at Asn127, the glycan at this position is not observed, because the presence of an N-glycan would create a severe steric hindrance with the fibronectin domain.

**Fig 3:**
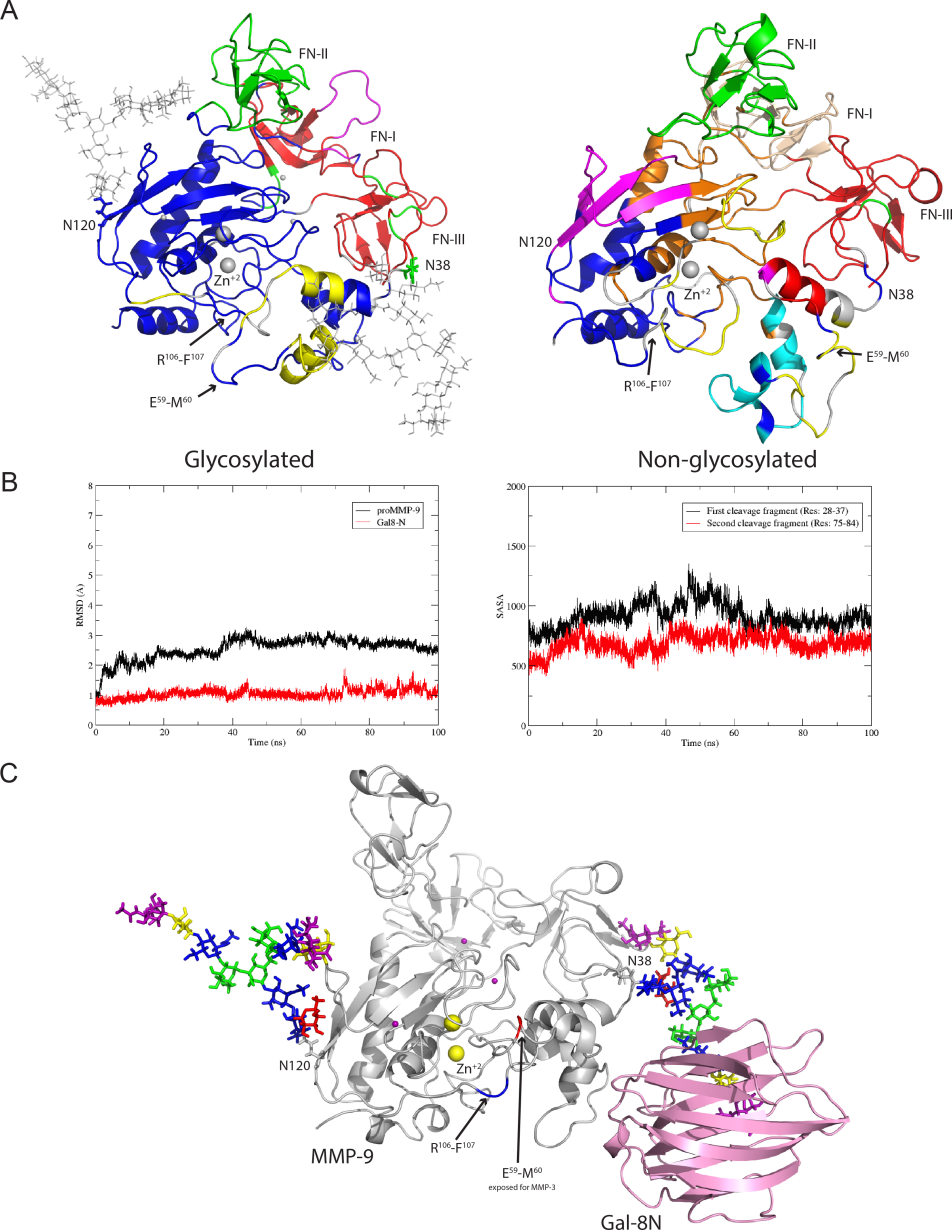
(A) Community analysis. Correlated motion of the sub-domains in the glycosylated and non-glycosylated forms of proMMP-9. Sub-domains that are moving together are represented in the same color. (B) Left and right panel represents the comparison of RMSDs of the pro and catalytic domains of glycosylated proMMP-9 and Gal-8N domain and the solvent-accessible surface area (SASA in Å^2^) of two proteolytic fragments for the complex composed of Gal-8N and glycosylated proMMP-9, respectively. (C) Structural model of the complex composed of Gal-8N and proMMP-9.

To understand the communication between groups of residues undergoing correlated motions in the glycosylated and non-glycosylated form of proMMP-9, we performed community network analysis [35]. For the glycosylated pro-MMP-9 (Fig 3A, left panel) we detected four main communities: 1) part of the prodomain and the catalytic domain (blue); 2) part of the prodomain (yellow); 3) the fibronectin domain II (FN-II), some residues of the prodomain and some fragments of secondary structures linking fibronectin repeats (green); and 4) the two fibronectin domains (FN-I and FN-III) (red). The residues belonging to each of these groups undergo correlated motion. In contrast, in the non-glycosylated proMMP-9, we observed seven well-defined communities colored accordingly (Fig 3A right panel). The community analysis revealed that in the glycosylated form of the proMMP-9 we observe consolidation of the structure into fewer subdomains exhibiting concerted movement within, i.e. the dynamics of the enzyme is less disorganized compared to non-glycosylated protein. Van den Steen *et al*. [36] attempted to assess the influence of N-glycosylation on activity of the MMP-9. However, the experimental techniques did not give conclusive information about the role of oligosaccharides in conformational dynamics. One of the problem was that obtaining fully N-deglycosylated form of the enzyme was not successful. It was demonstrated that only partially deglycosylated, but not fully deglycosylated, form maintained the activity, but the extent of associated conformational changes was not possible to determine. Thus, application of the structural experimental techniques, such as NMR, would be required to get detailed picture on conformational changes upon deglycosylation and to be able to compare such results with those obtained from our community analysis.

### Molecular dynamics simulation for the Gal-8N and glycosylated proMMP-9 complex

To understand the interaction of the Gal-8N and glycosylated pro-MMP-9 we performed 100 ns MD simulation for this complex. The appropriate RMSD plots for backbone atoms of the pro and catalytic domains of glycosylated proMMP-9 and for Gal-8N domain are presented in left panel of Fig 3B. They demonstrate that simulations are stable for the pro and catalytic domains of glycosylated proMMP-9 and Gal-8N domain throughout the entire MD simulations. We found that the terminal sialylated lactosamine of glycan at N38 fits well into the carbohydrate recognition domain (CRD) of Gal-8N domain (Fig 3C). The hydrogen bond analysis shows the Gal-8N domain, which has high affinity towards α2-3sialylated lactose, forms a hydrogen bond with sialic acid, galactose, and N-acetylglucosamine (Table 1). It is known, that the CRDs of Gal-8N and Gal-8C recognize sialylated lactosamine, and lactose or lactosamine, respectively [30, 31]. Also, Nishi *et al*. found that the CRD of Gal-8N interacts with glycosylated form of proMMP-9 and the CRD of Gal-8C interacts with integrin αM/CD11b and proMMP-9 [27]. The same study demonstrated that mutant R69H with an inactivated CRD of Gal-8N exhibited affinity for integrin αM/CD11b, which leads to retained adhesion activity and reduced affinity for proMMP-9. On the other hand, another mutant R233H with an inactivated CRD of Gal-8C retained affinity for proMMP-9 but did not bind integrin α, which leads to abolishing adhesion activation. In our MD simulations, we observe that the glycan at Asn38 by interacting with Gal-8N domain is prevented from forming interactions with amino acids at the surface of proMMP-9. In addition, we observe a conformational change in the first cleavage region that leads to the protection of the second cleavage, in the same way as has been observed in the simulation of glycosylated proMMP-9 structure without the Gal-8N. Right panel of Fig 3B represents calculated SASA values as a function of time for ten amino acids in the vicinity of cleavage regions. The SASA values are higher, meaning higher exposure, for the first cleavage region compared to the second one. Thus, the interactions between Gal-8N and glycosylated proMMP-9 facilitate the exposure of the first cleavage site for processing by MMP-3. Our structural findings are in agreement with the biochemical studies of Nishi *et al*. who concluded that MMP-3 mediated processing of glycosylated proMMP-9 is accelerated by Gal-8 through a ternary complex formation between glycosylated proMMP-9, Gal-8 and MMP-3. The schematic model of this ternary complex is shown in S5 Fig.

**Table 1:** Hydrogen bond analysis.

Donor	AcceptorH	Acceptor	Population
0SA_442:O1B	ARG_486:HH11	ARG_486:NH1	65.75
3LB_441:O4	ARG_510:HH21	ARG_510:NH2	64.09
3LB_441:O6	HIS_506:HE2	HIS_506:NE2	60.67
0SA_442:O1B	ASN_508:HD21	ASN_508:ND2	58.63
0SA_442:O9	ARG_500:HH11	ARG_500:NH2	16.56
4YB_440:O3	ASN_520:HD22	ASN_520:ND2	11.77
0SA_442:O1A	TYR_582:HH	TYR_582:OH	11.46

The results from hydrogen bond analyses of snapshots taken from last 50 ns MD simulation of Gal-8N-proMMP-9 Asn38 glycans complexes. Hydrogen bonds were calculated based on a geometric criterion (donor (D)-acceptor (A) distance < 3.0 Å, D-H-A angle > 120°). The table shows the percentage of the trajectory the hydrogen bonds were observed. The amino acids are represented by their standard three letter code and for the cabohydrates the GLYCAM nomenclature was used.

Analysis of MD simulation shows that the first proteolytic region is well exposed and available for the activation by MMP-3 when Gal-8N domain forms a complex with glycan at Asn38. Given the fact that the CRD of Gal-8C interacts with both integrin αM/CD11b and proMMP-9, it seems that these interactions may play a role in immobilizing proMMP-9 during the activation of proMMP-9 by MMP-3, which in turn may facilitate enzymatic processing.

## Methods

### Preparation of starting structure

The crystal structure of the truncated human pro-matrix metalloproteinase MMP-9 (gelatinase B; PDB ID: 1L6J) [37] was retrieved from the Protein Data Bank [38]. This structure is composed of the prodomain, catalytic domain and fibronectin inserts and it does not contain O-linker and hemopexin domains. This structure also has missing prodomain residues (R^56^VAEMRGESKS^66^) where the first MMP-3 mediated proteolytic site (E^59^-M^60^) is located. We modeled the missing region using structure of MMP-2 (gelatinase A; PDB ID: 1CK7) [39] as a template using the SWISS-MODEL server [40], which yields the RMSD of 0.416 Å between the model and the template. We use the Uniprot amino acid numbering for proMMP-9 (Uniprot ID: P14780) in this study.

We built the core fucosylated, sialylated complex (NeuAcα(1,2)-Galβ(1,4)-GlcNAcβ(1,2)-Manα(1,3)-[NeuAcα(1,2)-Galβ(1,4)-GlcNAcβ(1,2)-Manα(1,6)-]Manβ(1,4)-GlcNAcβ(1,4)-[Fucα(1,6)-]GlcNAcβ-Asn) glycan and then attached it to both the N-glycosylation sites (Asn38 and Asn120) using the Glycam webserver (http://www.glycam.org). The webserver uses GLYCAM force field for glycans for minimization and removes clashes with protein. The large, 3.051 Å value of RMSD difference between glycosylated and non-glycosylated proMMP-9 is mainly due to the structural differences in the prodomain region. We understand that under physiological condition oligosaccharides are highly flexible entities and a single static structure cannot represent their dynamic behavior. Thus, we performed MD simulation in explicit water to get a more complete understanding of the spatial and dynamic properties of this system and elucidate the role of glycosylation in two-step proteolytic activation.

In order to understand the accelerated activation of proMMP-9 by MMP-3 upon making a ternary complex with the N-domain of Gal-8, we retrieved the crystal structure of Gal-8 N terminal domain (Gal-8N) in complex with sialyllactosamine (PDB ID: 3VKO) [41]. Presently, no crystal structure of the Gal-8N protein interacting with N-glycan attached to proMMP-9 is available as a reference. Therefore, we built the starting model of the proMMP-9 Gal-8N complex by 3D-alignment with the terminal sialyllactosamine of Asn38 glycosylated proMMP-9 and sialyllactosamine Gal-8N complex and transferred the Gal-8N terminal domain into the binding site of the Asn38 glycosylated proMMP-9. All the catalytic histidine residues (His401, His405, and His411) were assumed to be neutral and were protonated at the Nδ-position. Initial structure of each glycosylated proMMP-9, non-glycosylated pro-MMP-9, and glycosylated proMMP-9—Gal-8N complex was prepared for MD simulations using the tleap module of the AMBER package. The preparation process involved addition of missing hydrogen atoms, counter ions required for electrostatic neutralization of the complex, and solvation box of TIP3P waters [42].

### Molecular dynamics simulations

MD simulations in explicit aqueous solvent were performed for each of the following systems: glycosylated proMMP-9, non-glycosylated proMMP-9, and glycosylated proMMP-9—Gal-8N complex. We used the AMBER force field ff99SB for the protein [43], while for carbohydrates parameters were taken from the GLYCAM06 force field [44]. The complexes were solvated in a rectangular box of TIP3P water using periodic boundary conditions. At the beginning, we performed energy minimization for all systems in order to remove initial unfavorable contacts made by the solvent using 10000 minimization cycles and keeping the protein backbone atoms restrained. Then, the systems were minimized keeping protein side chain atoms, counter ions and explicit water molecules unrestrained. Next, we run the unrestrained minimization with 10000 cycles of the whole system in each case. Secondly, all the systems were heated slowly from 5 to 300 K for 300 ps, followed by an equilibration step of 3 ns maintaining a constant temperature of 300 K and constant pressure of 1 atm. For the catalytic His-Zn interaction distance restraints of <4 Å between atoms His401(Nε), His405(Nε), His411(Nε) and Zn were applied in order to stabilize the interaction during the equilibration period and to force catalytic His residues to maintain a stable interaction with the Zn ion. We chose to restrain distances between Nε atoms and the Zn ion because they were found to be closest to each other in the crystal structure. Finally, production phase of the MD simulations was performed at 300 K and constant pressure of 1 atm for additional 500 ns using a 2-fs time step for glycosylated proMMP-9, non-glycosylated proMMP-9, and 100ns for glycosylated proMMP-9 Gal-8N complex. We performed two independent molecular dynamics simulations, 500ns long each, for glycosylated proMMP-9 initiated from different distributions of atomic velocities. Additionally, we built a model for the ternary complex of proMMP-9, Gal-8N and MMP-3 to illustrate hypothetical structural interactions between the binding partners, however we did not perform the actual MD simulations for this model. During the simulations, the SHAKE algorithm was turned on and applied to all hydrogen atoms and the particle-mesh Ewald method was used for treating the electrostatic interactions. A cutoff of 10 Å was used for non-bonded interactions. Minimization, equilibration, and production phases were carried out by the PMEMD.cuda_SPFP module of AMBER 12 [45].

### Trajectory analysis

The analysis of MD simulations was performed using the ptraj module of AmberTools 12 [46] which was used for the superimposition of the trajectory frames and to strip water and counter ions from the trajectory for visualization with VMD. The root means square deviation (RMSD), root mean square fluctuation (RMSF), B-factor, and dssp modules were used to analyze each frame of the MD production runs to determine the average overall fluctuation, conformational fluctuation of each residue and secondary structure. The solvent-accessible surface area (SASA) and hydrogen bond analysis modules of ptraj were used to analyze accessible surface area of ten amino acid regions in the vicinity of cleavage sites and hydrogen bond interaction between glycans and proteins, respectively. To identify protein segments involved in correlated motions we performed community network analysis using Bio3D package [47]. All figures were made using either the PyMOL Molecular Graphics System (DeLano Scientific, Palo Alto, CA), VMD or Xmgrace software.

## Supporting information

S1 Fig Schematic representation of the proMMP-9 domains. The prodomain, the catalytic domain, and the fibronectin domain are shown in blue, red, and green color respectively, catalytic Zn^+2^ (yellow sphere) is labelled.
(PNG)

S2 Fig Comparison of RMSF plots for all amino acids of the glycosylated and the non-glycosylated form of proMMP-9. (PNG)

S3 Fig Schematic model of the glycosylated proMMP-9 (grey) and MMP-3 (green). (PNG)

S4 Fig **Secondary structure analysis of the 500ns trajectory for: (A) the glycosylated and (B) the non-glycosylated form of the proMMP-9 prodomain.** The first (E^59^-M^60^) and second (R^106^-F^107^) cleavage sites are marked.
(PNG)

S5 Fig Schematic model of the glycosylated proMMP-9 (grey), Gal-8N (pink) and MMP-3 (green) ternary complex. The first (E^59^-M^60^) and second (R^106^-F^107^) cleavage sites are marked with red and blue lines, respectively.
(PNG)
