# Supplementary figures and images for: Role of N-glycosylation in activation of proMMP-9. A molecular dynamics simulations study
Source: PLoS One. 2018 Jan 12;13(1):e0191157. doi: 10.1371/journal.pone.0191157 (PMC5766141; doi:10.1371/journal.pone.0191157)

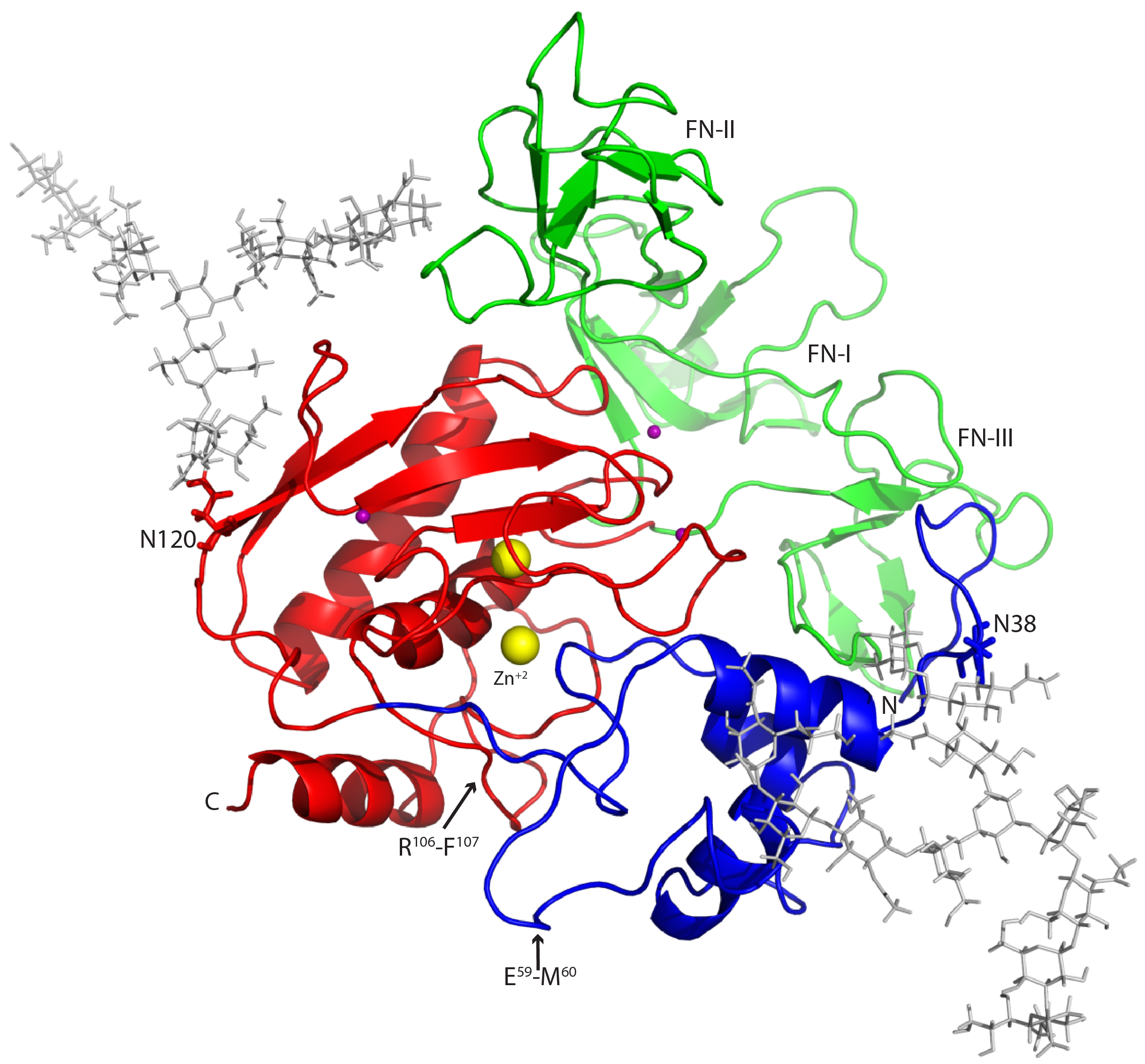

Supplement: S1 Fig — The prodomain, the catalytic domain, and the fibronectin domain are shown in blue, red, and green color respectively, catalytic Zn+2 (yellow sphere) is labelled. (PNG) [file pone.0191157.s001.png]

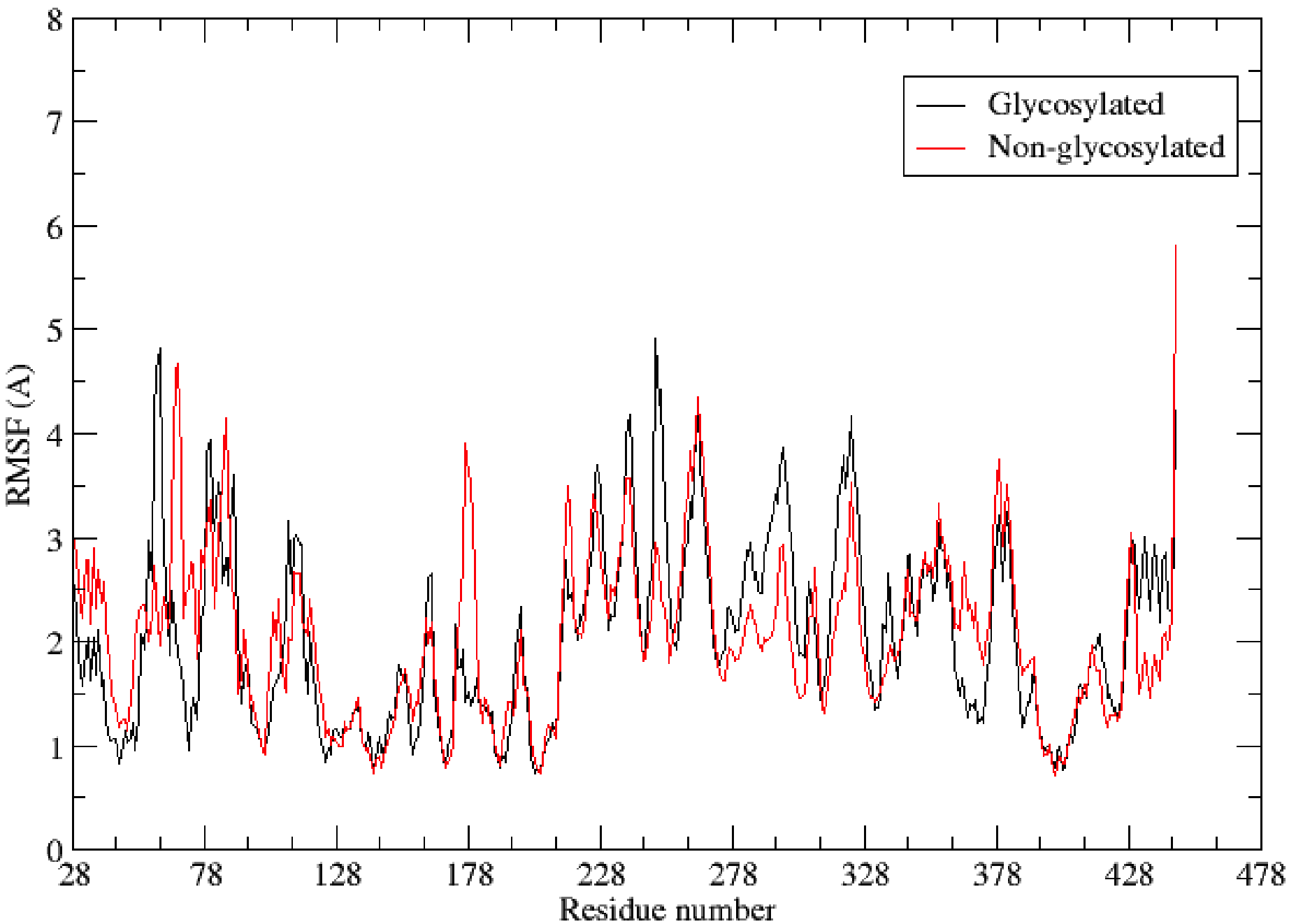

Supplement: S2 Fig — (PNG) [file pone.0191157.s002.png]

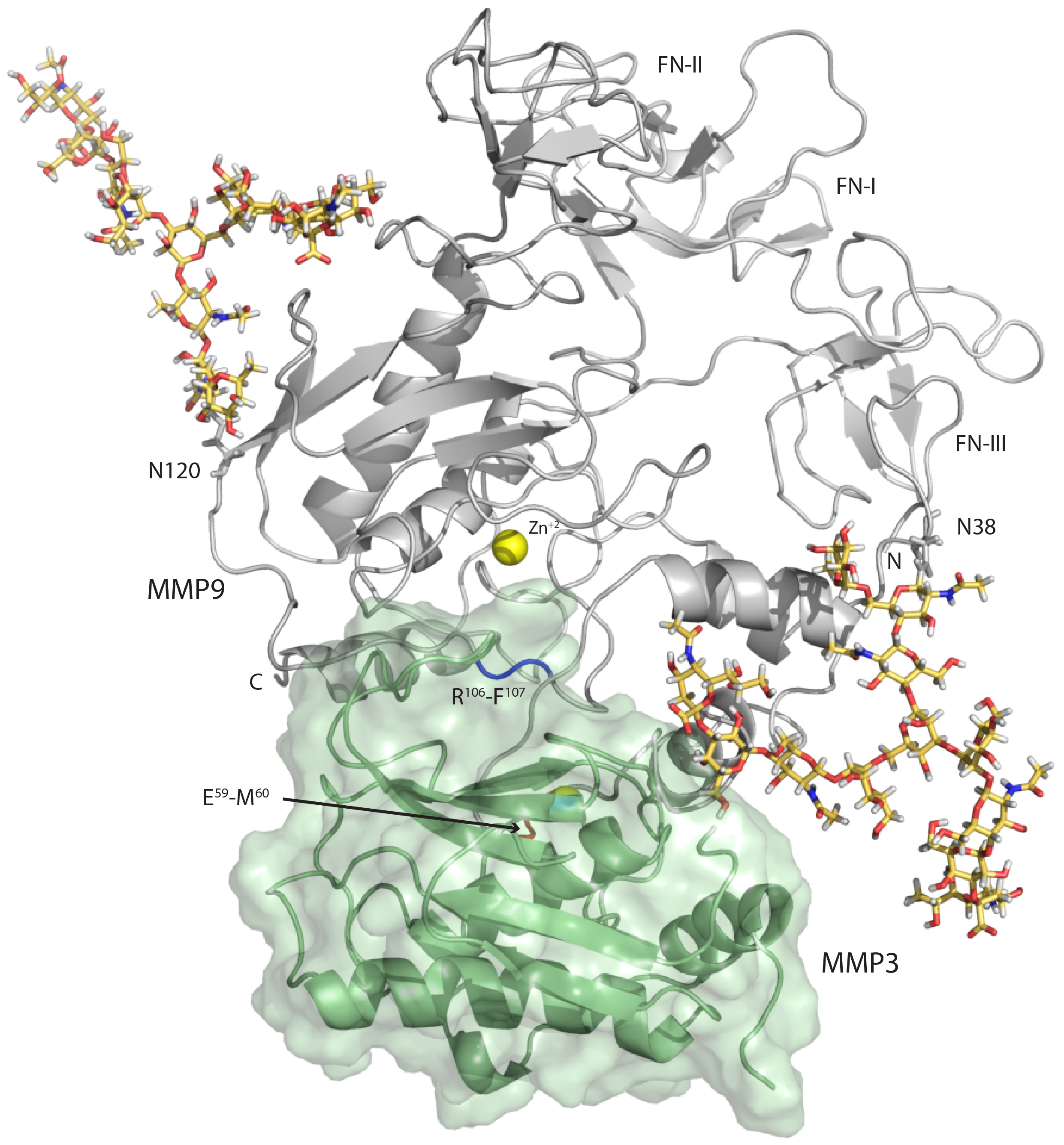

Supplement: S3 Fig — (PNG) [file pone.0191157.s003.png]

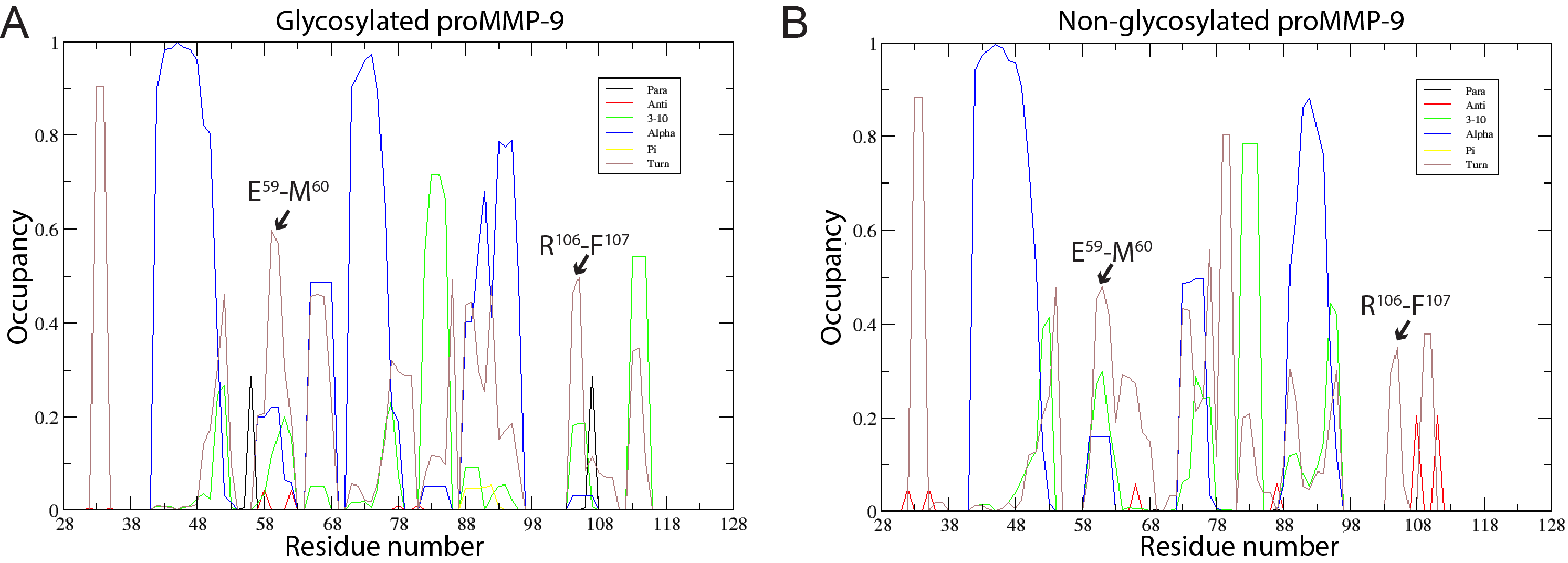

Supplement: S4 Fig — Secondary structure analysis of the 500ns trajectory for: (A) the glycosylated and (B) the non-glycosylated form of the proMMP-9 prodomain. The first (E59-M60) and second (R106-F107) cleavage sites are marked. (PNG) [file pone.0191157.s004.png]

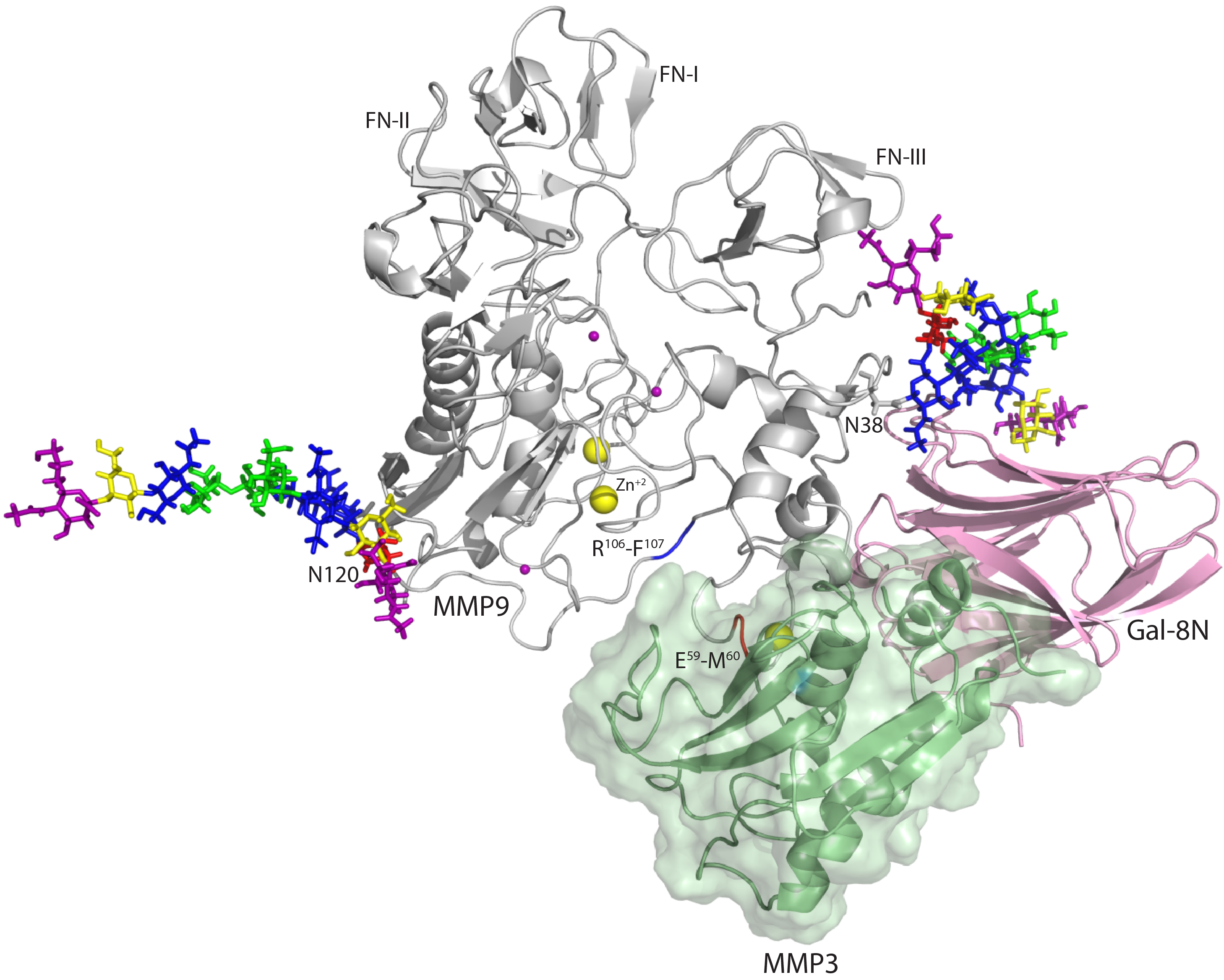

Supplement: S5 Fig — The first (E59-M60) and second (R106-F107) cleavage sites are marked with red and blue lines, respectively. (PNG) [file pone.0191157.s005.png]
